# Amino Acid Deficiency Secondary to Continuous Venovenous Hemofiltration in Acute Decompensation of Organic Acidemias: An Anabolic Dead End?

**DOI:** 10.1097/CCE.0000000000001413

**Published:** 2026-08-03

**Authors:** Chloé Grosyeux, Noémie Cammiciotto, Elise Jeannesson, Eva Feigerlova, Valentine Villada, Camille Wicker, Michael Levy, Manuel Schiff, Apolline Imbard, David Coelho, François Feillet, Arnaud Wiedemann

**Affiliations:** 1 Pediatric Nephrology Department, University Hospital of Nancy, Nancy, France.; 2 Pediatric Department, Reference Center of Inborn Errors of Metabolism (ORPHA67872), University Regional Hospital Center of Nancy, INSERM UMRS 1256 NGERE—Nutrition, Genetics, and Environmental Risk Exposure, Nancy, France.; 3 Division of Biochemistry, Department of Molecular Medicine, University Hospital of Nancy, Nancy, France.; 4 Pediatric Inherited Metabolic Diseases Department, University Hospital of Strasbourg—Filière G2M, Strasbourg, France.; 5 Pediatric Intensive Care Unit, University Hospital of Strasbourg, Strasbourg, France.; 6 Pediatric Department, Reference Center for Inborn Error of Metabolism, Hôpital Necker-Enfants Malades, APHP and Université Paris Cité, Paris, France.; 7 Département Médicaments et Technologies pour La Santé (DMTS), Paris-Saclay University, CEA, Gif-sur-Yvette, France.; 8 Inserm UMRS_1163, Institut Imagine, Paris, France.; 9 Pediatric Intensive Care Unit, University Regional Hospital Center of Nancy, Nancy, France.; 10 Research Center, Sainte-Justine Hospital, University of Montreal, Montreal, QC, Canada.

## Abstract

**OBJECTIVE:**

To evaluate the impact of continuous renal replacement therapy (CRRT) on plasma amino acid (AA) concentrations in patients with acute metabolic decompensation of organic acidemias (OAs), and to explore whether AA supplementation during CRRT may mitigate AA depletion.

**DESIGN:**

Multicenter retrospective observational study.

**SETTING:**

PICUs managing acute metabolic decompensations of OAs.

**PATIENTS OR SUBJECTS:**

Patients with confirmed OAs who underwent CRRT for severe acute metabolic decompensation.

**INTERVENTIONS:**

CRRT was initiated according to current guidelines in cases of severe decompensation. Standard metabolic management included high caloric intake through carbohydrates and lipids with temporary protein withdrawal (24–48 hr). In one patient, AA supplementation (2 g/kg) during CRRT combined with thiamin and citrate (anaplerotic therapy) was administered.

**MEASUREMENTS AND MAIN RESULTS:**

Plasma AA concentrations were measured before and after CRRT in nine patients with a median age of 21 days (interquartile range [IQR], 3–570 d). Quantitative variables are expressed as medians (IQR, 25th–75th). Before CRRT, 31% (95% CI, 24–39) of plasma AAs were below the normal range compared with 69% (95% CI, 59–77) after CRRT (*p* < 0.0001). A significant increase in lactatemia was observed following CRRT, without evidence of organ failure: median 2.2 mmol/L (IQR, 1.3–2.3) before CRRT vs. 5.3 mmol/L (IQR, 3.2–7.1) after CRRT; Hodges-Lehmann median difference +3.2 (95% CI, 0.8–5.6; *p* = 0.0065). In the patient receiving AA supplementation with anaplerotic therapy, plasma AA status improved markedly, with 65% of AAs below normal before CRRT vs. 15% after CRRT.

**CONCLUSIONS:**

In acute decompensated OAs, CRRT performed without protein supplementation, as currently recommended, significantly reduces total plasma AA concentrations and may impair restoration of anabolism. AA infusion during CRRT could help preserve or restore protein anabolism. Prospective studies are needed to assess the safety and efficacy of AA supplementation during CRRT, with or without anaplerotic therapy, in this setting.

**KEY POINT**

**Question:** Does continuous renal replacement therapy (CRRT) performed during acute decompensation of organic acidemias (OAs) further decrease plasma amino acid (AA) concentrations and potentially impair restoration of anabolism?

**Findings:** In this multicenter retrospective study of nine patients, the proportion of plasma AAs below the normal range increased significantly after CRRT (31% before vs. 69% after). Lactatemia also increased after CRRT. In one patient, AA supplementation during CRRT improved plasma AA status.

**Meaning:** CRRT without protein supplementation may aggravate AA depletion in decompensated OAs; AA infusion during CRRT warrants prospective evaluation.

Organic acidemias (OAs) are a group of rare inherited metabolic disorders characterized by defect in branched-chain amino acid (AA) catabolism (1). Among them, propionic acidemia (PA) (OMIM Number 606054) and methylmalonic acidemia (MMA) (OMIM Number 251000) result from disruptions in propionate catabolic pathway. PA is caused by a deficiency of propionyl-CoA carboxylase, whereas MMA (OMIM Number 251000) arises from a deficiency of methylmalonyl-CoA mutase or from defects in its cofactor adenosylcobalamin (2, 3).

In both disorders, acute metabolic decompensation episode (MDE) is associated with the accumulation of organic acids (methylmalonic or propionic and their metabolites), which results in a severe high-anion gap metabolic acidosis. The increased anion gap is accounted for not only by these accumulated metabolites but also by ketone bodies, the latter arising as a consequence of secondary inhibition of ketolysis induced by the toxic metabolites (4). The diagnosis can be made by observing high metabolite values in urine (MMA and PA) and/or plasma (MMA) or carnitine derivatives (propionylcarnitine [MMA and PA] and C4-DC [MMA]) (5). Hyperammonemia in MMA and PA primarily results from inhibition of the urea cycle. Accumulated propionyl-CoA and methylmalonyl-CoA reduce synthesis of *N*-acetylglutamate (NAG), the essential activator of carbamoyl phosphate synthetase I, and deplete tricarboxylic acid (TCA) cycle intermediates needed to supply aspartate for nitrogen disposal. Mitochondrial dysfunction and catabolic stress further compromise energy-dependent ammonia detoxification, also explaining in part hyperammonemia in MMA and PA (4). NAG depletion and subsequent hyperammonemia may be at least partially corrected by oral carglumic acid, a structural analog of NAG (6–10).

In cases of MDE, prompt management of hyperammonemia and ketoacidosis are critical, as neurologic outcomes are closely linked to the rapidity of intervention (11, 12). The primary goal of treatment is to restore an anabolic state. This is achieved by administering high caloric intake from carbohydrates and lipids without protein or AAs intakes (13). In the most severe cases, with neurologic symptoms, highest plasma ammonia levels (e.g., above 500 µmol/L in neonates) or refractory acidosis (pH < 7.10), continuous renal replacement therapy (CRRT) is indicated. CRRT facilitates the rapid correction of both hyperammonemia and associated metabolic disturbance responsible of acidosis (methylcitrate, propionate, etc) (14, 15). However, CRRT results in the unintended loss of AA through the ultrafiltrate (16, 17).

This study aimed to investigate the changes in plasma AA concentrations during CRRT for metabolic decompensation in patients with OA, and to explore the potential link between AA profiles and the ability to restore an anabolic status allowing a resumption of protein synthesis.

## MATERIALS AND METHODS

### Study Population

We conducted an observational, retrospective, multicenter study from 2015 to 2024 at University Hospitals of Nancy (France) and Strasbourg (France) including data from nine patients with hyperammonemia due to MDEs in OAs (at diagnosis and during follow-up) and treated by CRRT. Patients were included only if they experienced an acute metabolic decompensation and met the clinical and/or biological criteria for CRRT initiation (hyperammonemia > 500 µmol/L, neurologic symptoms, or metabolic disturbance responsible of acidosis with pH less than 7.10 [15]). All patient excepted patient 1 were managed according to our protocols extracted from national standardized emergency protocol (18). In case of OA decompensation, the protocol purpose to give high energy intake (120% of basal metabolic requirements) mainly provided by high dose dextrose infusion (according to weight and age) and lipids, aimed at reversing catabolism. Proteins intakes were stopped. Sodium benzoate (250 mg/kg/d), carglumic acid (100 mg/kg/d), and levocarnil infusion (100 mg/kg/d) were administrated. No anaplerotic therapy used to be prescribed, except for patient 1, who presented a severe lactic acidosis despite recommended organ supply therapies. In this situation, we use citric acid and thiamin. The ethics committee of the University Hospital of Nancy approved the July 10, 2025, this study called “HyperAAmonniémie et épuration extra rénale” (2024PI169-626). Whether procedures were followed in accordance with the ethical standards of the responsible committee on human experimentation (institutional or regional) and with the Helsinki Declaration of 1975.

### Study Aims and Outcomes

The primary aim of this study was to evaluate the evolution of plasma AA concentrations during CRRT in decompensated OA. Secondary aims were to evaluate the evolution of lactatemia, pH, and ammonia during CRRT in decompensated OA.

### Data Collected for This Study

The following administrative and clinical data were obtained by electronic chart review: date of birth; initial pathology; sex; date of CRRT; CRRT time; date of diagnosis; and laboratory parameters (AA plasma concentrations; ammonia; lactatemia; pH) before, during, and after CRRT (i.e., before: just before starting CRRT; during: whereas CRRT was working; and after: the next day or just after [maximum 3 d after] CRRT withdrawal). Indication of CRRT was also collected.

### Ratio Calculation

Ratios were calculated as defined by Whitehead et al (19): first the mean value for each individual AA was calculated by averaging all patients’ values before and after CRRT. The sum of nonessential AA was divided by the sum of essential AA. We also calculated each ratio at the individual level. We calculated the ratio of each individual AA to its lower normal value, and finally, the median ratio value of all essential and nonessential AA ratios.

### Continuous Renal Replacement Therapy

Patients received CRRT through hemodiafiltration (continuous venovenous hemodiafiltration, CVVHDF) using a protocol standardized across both tertiary centers. The machines used for hemofiltration were Prismax or Prismaflex (Baxter, Baxter International Inc., Deerfield, IL). Only bicarbonate-buffered fluids were used. Anticoagulation was performed with unfractionated heparin exclusively. The choice of anticoagulation was at the discretion of the clinician. In all cases, we prescribed an ultrafiltrate rate (Quf) of 35 mL/kg/hr, with an adaptative blood flow rate (Qb) to target a filtration fraction of 20–25%.

### Statistical Analysis

All quantitative variables are described as medians (apart from the calculating ratios) and percentiles (interquartile ranges [IQR], 25th–75th percentiles), and qualitative variables are reported as percentages and 95% CIs. We compared quantitative variables using the Kruskal-Wallis test. We compared qualitative variables using the chi-square test. To maximize the representativeness of this rare disease cohort, we performed an available case analysis; all documented AA profiles were included in the descriptive statistics, regardless of whether paired pre- and/or post-CRRT data were available for every individual. All statistical analyses were conducted using GraphPad Prism, Version 8.4.3 (GraphPad Software [a Dotmatics company], Boston, MA) based on a two-sided type I error with an alpha level of 0.05.

## RESULTS

### Description of the Studied Population

Between 2015 and 2024, nine patients were included and analyzed in this study. Baseline characteristics of the patients are reported in **Table 1** and **Table S1** (https://links.lww.com/CCX/B638). The median age was 21 days (IQR, 3–570), including 44% (4/9) of males. The median ammonia at CRRT initiation was 470 µmol/L (IQR, 146–543) for all patients. In six patients, CRRT was initiated for hyperammonemia, and in four patients for severe metabolic acidosis (Table 1). Median lifespan of filter was 420 minutes (IQR, 270–2079). A total of five patients presented with an initial MDE at the time of diagnosis, whereas four patients experienced an MDE during subsequent follow-up (Table 1). Eight patients were without dietary protein at the time of CRRT as recommended (median ammonia at CRRT initiation was 488 [IQR, 153–610]). The median *z* score body mass index (BMI) was –2.4 for the group already followed at the time of decompensation, and –1.6 for the group with initial decompensation. One patient with PA (patient 1) and multiple organ failure was supplemented by a continuous infusion of 2 g/kg of AA (primene 10%, AA injection solution) along with oral sodium citrate and thiamin. This occurred 20 hours after CRRT began, and there was no period of protein restriction.

**TABLE 1.** Main Demographic and Baseline Characteristics

Demographics		
Age at diagnosis (d), median (IQR)	4 (3–21)	
Age at CRRT (d), median (IQR)	21 (3–570)	
Male sex, *n*/*N* (%)	4/9 (44)	
CRRT by CVVHDF, *n*/*N* = 9/9		
Initial decompensation (at the time of diagnosis), *n*/*N* (%)	5/9 (56)	
Metabolic decompensation episode during follow-up, *n*/*N* (%)	4/9 (44)	
Reasons of CVVHDF, *n*/*N* (%)		
Hyperammonemia	6/9 (67)	
Ketoacidosis	4/9 (44)	
Neurologic disorder	3/9 (33)	
Mixed	3/9 (33)	
Days under dialysis, median (IQR)	2 (1.5–3.4)	
Filter lifespan (min), median (IQR)	470 (288–2058)	
Type of disease, *n*/*N* (%)		
Propionic academia	4/9 (44)	
Methylmalonic acidemia	5/9 (56)	
Laboratory parameters, median (IQR)	Before dialysis	After dialysis
Aspartate aminotransferase (UI/L)	30 (29–50)	31 (26–85)
Alanine aminotransferase (UI/L)	77 (28–98)	79 (31–81)
Patients without protein supplementation, *n*/*N* = 8/9		
Ammonia (µmol/L)	488 (153–610)	63 (40–85)
Lactate (mmol/L)	2.2 (1.3–2.3)	5.3 (3.2–7.1)
pH	7.197 (7.104–7.254)	7.338 (7.237–7.360)
Supplemented patient^a^, *n*/*N* = 1/9		
Ammonia (µmol/L)	146	38
Lactate (mmol/L)	4.3	3.7
pH	6.988	7.412

*n*/*N* = number of cases, CRRT = continuous renal replacement therapy, CVVHDF = continuous venovenous hemodiafiltration, IQR = interquartile range.

a Supplemented with thiamin, citrate, and 2 g/kg of amino acid (primene 10%).

### CRRT in MDEs Without Protein Administration Provoked a Significant Deprivation of Plasma AA

**Figure 1.**
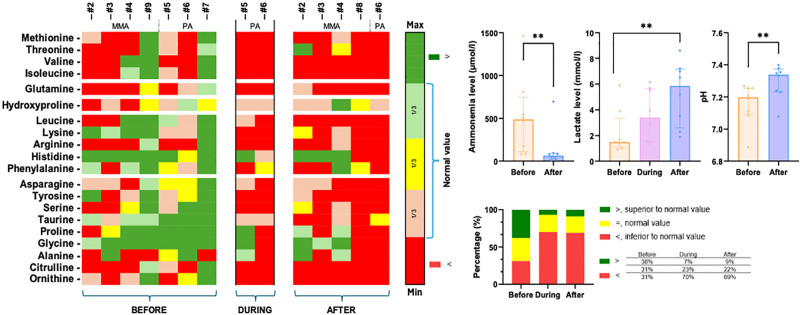
Deprivation of amino acids during continuous renal replacement therapy in decompensated organic acidurias without protein supplementation with an increase of lactate blood level and a normalization of ammonia and pH. MMA = methylmalonic aciduria, PA = propionic acidemia, Max = maximum, Min = minimum.

Among the eight patients without protein supplementation, among all the AA, blood concentrations were below normal in 31% (95% CI, 24–39) before CRRT vs. 69% (95% CI, 59–77) after CRRT (*p* < 0.0001) (**Fig. 1**). The individual time courses of each AA are shown in **Figure S1** (https://links.lww.com/CCX/B638).

Among patients with initial decompensation and without protein supplementation, all the AA were below normal in 33% (95% CI, 23–46) before CRRT vs. 58% (95% CI, 46–70) after CRRT (*p* < 0.0049). Among the followed patients without protein supplementation, all the AA were below normal in 37% (95% CI, 26–49) before CRRT vs. 85% (95% CI, 71–93) after CRRT (*p* < 0.0001). There was no significant difference between the initial decompensation group and the followed group (*p* = 0.75).

For the essential AAs, 37% (95% CI, 26–49) were below normal before CRRT vs. 76% (95% CI, 61–86) after (*p* < 0.0001). For the nonessential AAs, 26% (95% CI, 18–37) were below normal before CRRT vs. 64% (95% CI, 50–75) after CRRT (*p* < 0.0001).

### Deprivation of Plasma AA is Associated With an Increase of Lactate Blood Level and a Normalization of pH and Ammonia

**Figure 2.**
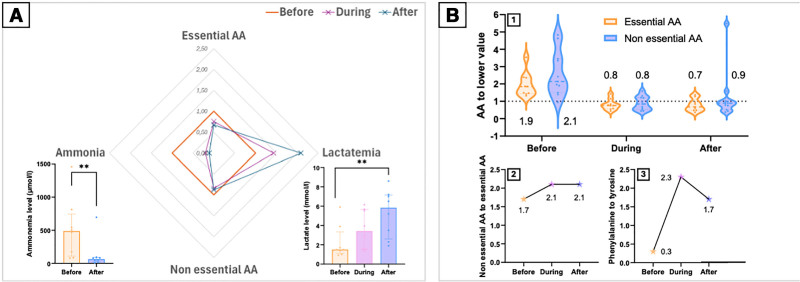
The multiple effects of continuous renal replacement therapy (CRRT) in decompensated organic acidurias without protein supplementation. **A**, Radar graph normalized to values before CRRT (*orange*), which serve as the reference baseline (value = 1). The values during (*purple*) and after (*blue*) CRRT are expressed as proportions relative to this baseline. The plot displays essential amino acids (*top*), lactatemia (*right*), nonessential amino acids (*bottom*), and ammonia levels (*left*). *Bar plots* show a significant decrease in ammonia levels (*p* < 0.01) and a significant increase in lactatemia (*p* < 0.01) after CRRT. **B**, Amino acid (AA) ratios were calculated following the method described by Whitehead et al (17): 1) The mean of each individual AA was computed across all patients and then divided by its lower reference value. The median of these ratios was calculated separately for essential (*orange*) and nonessential (*blue*) AAs. 2) The ratio of the mean of nonessential AAs to essential AAs across all patients was calculated for each condition (before, during, and after CRRT). 3) The phenylalanine-to-tyrosine ratio was calculated too.

Among eight patients without protein supplementation, the median lactatemia was 2.2 mmol/L (IQR, 1.3–2.3) before and 5.3 mmol/L (IQR, 3.2–7.1) after CRRT (Figs. 1 and **2**). Lactatemia was significantly higher after CRRT than before CRRT, with a difference between medians (Hodges-Lehmann) of + 3.2 mmol/L (95% CI, 0.8–5.6; *p* = 0.0065).

pH level increased significantly from 7.197 (IQR, 7.104–7.254) before CRRT to 7.338 (7.237–7.360) after CRRT (Fig. 1). pH was significantly higher after CRRT than before, with a difference between medians of +0.13 (*p* = 0.046).

Ammonia levels decreased significantly from 488 µmol/L (IQR, 153–610) before CRRT to 63 µmol/L (IQR, 40–85) after CRRT (Figs. 1 and 2). Ammonia was significantly lower after CRRT than before CRRT, with a difference between medians of –416 µmol/L (95% CI, –730 to –33; *p* = 0.015).

### There Was No Deprivation of Plasma AA and No Increase of Lactatemia Blood in the Supplemented Patient 1

**Figure 3.**
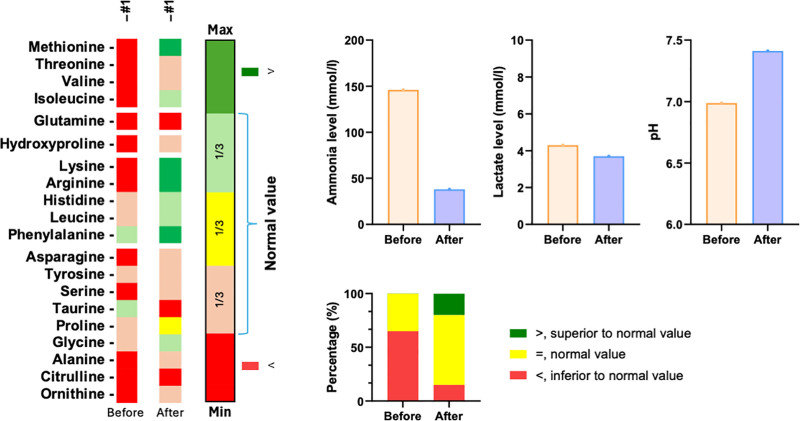
Effect of protein supplementation inpatient 1. Continuous renal replacement therapy. Max = maximum, Min = minimum.

In the patient supplemented with 2 g/kg of AA, but also thiamin and citrate, AA concentrations were below normal for 65% of AA before and 15% after CRRT (**Fig. 3**). Lactatemia was 4.3 mmol/L before and 3.7 mmol/L after CRRT. pH level was at 6.99 before and 7.41 after CRRT. Ammonia level decreased from 146 µmol/L to 38 µmol/L after CRRT.

### CRRT Effect on Essential AA and Nonessential AA

Among the eight patients without protein supplementation, regarding essential AA, the median ratio to lower normal value was 1.9 (IQR, 1.5–2.3) before and 0.7 (IQR, 0.5–0.9) after CRRT. The ratio observed after CRRT was significantly lower than before CRRT, with a difference between medians of –1.0 (95% CI, –1.7 to –0.5; *p* ≤ 0.0001) (Fig. 3). Regarding nonessential AA, the median ratio to lower normal value was 2.1 (IQR, 1.7–3.4) before and 0.9 (IQR, 0.6–1.0) after CRRT. The ratio observed was significantly lower after CRRT than before CRRT, with a difference between medians of –1.3 (95% CI, –2.6 to –0.6; *p* = 0.0024) (Fig. 3).

The ratio of nonessential to essential AA was 1.72 before and 2.13 after CRRT. At the individual level (per patient), the ratio ranged from 1.40 to 5.59 before CRRT and from 1.14 to 2.91 after (no significant). The median was 1.93 before CRRT and 2.03 after, with a difference between medians of + 0.34 (95% CI, –1.21 to 1.24; *p* = 0.7551). The ratio of phenylalanine to tyrosine was 0.31 before and 1.72 after CRRT (no significant) (Fig. 3).

## DISCUSSION

In cases of severe MDE in OAs, CRRT is required and typically achieves rapid correction of blood pH and ammonia levels. However, we observed a marked reduction in plasma AA concentrations, accompanied by an increase in lactatemia. The decline was most pronounced for essential AAs, potentially impairing protein synthesis and delaying restoration of an anabolic state, thereby raising questions about the current practice of withholding AA supplementation during CRRT in severe cases. Loss of AA in case of CRRT or hemodialysis (HD) is well documented in ICUs or nephrology units. Maxvold et al (20) report in their study an AA loss in CRRT associated with a negative nitrogen balance responsible for a catabolic state. This loss was also reported in case of continuous low-efficiency dialysis (21). In chronic dialysis, this AA loss is known to be associated with a significant decrease in plasma AA concentrations (22). As a result, this AA leak induce a hypercatabolism state, which is associated with a loss of muscle mass (cachexia) (23). In case of acute renal failure or chronic renal disease, increasing dietary protein prescriptions or introducing AA supplementation may avoid this situation. In fact, nitrogen balance is achieved with a standard protein intake of 1.5 g/kg/d and a resting energy expenditure of 120% of calories (20). These target values are recommended by the latest guidelines for patients on HD (24). In OA, AA metabolism is disturbed, leading to elevated glycine levels and decrease of glutamine, but also of some of essential AA (i.e., isoleucine and valine). A pool of AAs is required to maintain an anabolic state, enabling protein synthesis essential for proper cellular function. In the case of an AA deficiency—particularly of essential AAs—a limiting AA may impair protein synthesis, thereby disrupting cellular processes (25, 26).

In inborn errors of metabolism (i.e., M) with acute decompensation episode, recommended first line treatment are high carbohydrate and lipid intakes to stimulate anabolism associated to protein free diet. Treatment may be upgraded by the use of scavengers, vitamins, and for the more severe cases CRRT without protein supplementation (15, 27). As we observed, this therapeutic strategy is associated with significant plasma AA depletion. The implementation of a filtration dose of 35 mL/kg/hr and high-flux membranes, although necessary for rapid ammonia clearance, likely contributed to the significant AA loss observed in our study. In decompensated OAs, the four AA precursors of propionate (methionine, threonine, valine and isoleucine) are decreased and remain low after CRRT. Another example is glutamine, which blood levels are dramatically low after CRRT. As glutamine may serve as an essential AA for anaplerosis in OAs (28), aggravation of its deficiency could exacerbate mitochondrial dysfunction. In one patient with acute decompensation, we voluntarily infused AA at 2 g/kg and noticed a different profile, with less plasma AA deprivation. To note, glutamine remained under normal value in this patient.

AAs are fundamental for anabolism, furthermore essential AA (29). Many studies have reported that restricting or depriving the diet of essential AA causes profound alterations in energy balance (30). These clinical situations are also described in severe malnutrition cases of kwashiorkor, where AA ratio shows a critical deficiency in essential AA (19, 31, 32) due to a protein deficiency and responsible for severe energetic metabolic failure (33–35). Our study showed an imbalance between essential and nonessential AA. The ratio of nonessential to essential AA increased after CRRT, whereas the ratio of both essential and nonessential AA to their lower value significantly decreased after CRRT. These findings suggest that essential AA tend to decrease relatively more than nonessential AA, as described in severe malnutrition. AA deprivation increases catabolic status, responsible for acceleration of protein degradation similar to cachectic condition (36). AA deprivation, particularly branched-chain AA (leucine, isoleucine, and valine), is also associated with a metabolic stress that reduces general messenger RNA translation (37). Our assessment of nutritional status was primarily based on BMI *z*-scores. More specific markers of protein turnover (e.g., pre-albumin, or albumin) were not available due to the retrospective and emergency nature of the study.

Although the exact pathophysiology of OA remains incompletely understood, disturbance in energy metabolism may take an important place (8). Multiomics analysis have identified dysfunction of the citric acid cycle due to mitochondrial alteration in MMA (38). The TCA cycle is a metabolic pathway that supplies cells with energy through aerobic respiration. Anaplerotic pathways regenerate TCA cycle intermediates to maintain flux under homeostasis from either the dietary supply of AA or catabolism (39–41). Cataplerosis, the loss of intermediates from the TCA cycle are thought to play a key role, and anaplerotic therapies are a promising approach. These approaches aim to replenish TCA cycle intermediates by providing substrates to support energy production and metabolic homeostasis (28, 42). TCA dysfunction leads to pyruvate accumulation, which is redirected to lactate dehydrogenase, resulting in elevated lactate levels. We reported a significant increase of lactate blood value at the end of therapy, whereas ammonia decreased, pH normalized, and no patients presented any other organ failure. Furthermore there was no hyperglycemia after CRRT (blood glucose is filtered in dialysis) which could explain this hyperlactatemia. This increase of lactate blood level is known and reported in other case (43), but is not reported after dialysis session in other clinical situation. To note, the molecular weight of lactate is 90 g/mol and is therefore filtered. The rebound therefore suggests that production exceeds clearance capacity when dialysis is stopped. Furthermore, this lactate rebound following CRRT discontinuation, but its absence in the supplemented patient, suggests that this phenomenon is not merely the natural course of the metabolic crisis. Instead, it likely reflects the cessation of the high metabolic clearance provided by CRRT, whereas the underlying catabolic state, responsible for lactate production, remained partially unresolved. Indeed, pathophysiology of organic aciduria remain poorly understood. In urea cycle disorders, enzyme deficiency lead to hyperammonemia and so increase synthesis of glutamine by hepatocytes to limit ammonia toxicity (44). In cases of OA, we observed low glutamine blood concentration despite increase of ammonia blood concentration because of carbamoyl phosphate synthetase although decrease of acetyl-CoA and glutamate pool (28). Defect of Krebs products is a serious lead to explain pathophysiology of OA (8 forny), and so a mitochondrial cataplerotic state might be associated, responsible of pyruvate conversion into lactate to produce energy rather than oxidative phosphorylation (38). According to this theory, increase of lactate is not only the result of accumulated toxic metabolite, but one marker of persistent metabolic dysfunction due to Krebs cycle cataplerosis. This observation underlines the need of AA to restore anabolic synthesis, in patients with OA supply by CRRT or HD.

Certain limitations of the present study on an orphan disease must be acknowledged. Notably, the limited number of patients included may not be fully representative of the larger population. Nevertheless, OA has a prevalence of 1/100,000, and in our study region, there are 50,000 births per year, representing approximately one to two cases per year, consistent with our cohort. Furthermore, our study focuses on a specific high acuity subgroup of patients. Although the recruitment in one tertiary referral center between two hospitals offer an interesting overview of this rare condition, but description of patients in PICU introduce a selection bias toward more severe cases, requiring renal replacement therapy for metabolic crisis. Finally, due to the small sample size, we performed an available case analysis rather than a complete case analysis, which may have introduced selection bias.

In ICUs, CRRT, especially high-dose CVVHDF, is recommended in cases of acute decompensation and hyperammonemia (15, 26). This therapy also caused severe AA loss, which could alter restoration of an anabolic status. An AA infusion may limit this condition, and improve metabolic status as suggested by our experience. Further studies are necessary to explore anaplerotic management, like infusion of AA, in cases of acute metabolic decompensation in OA, to improve energy metabolism and outcomes.

## Supplementary Material
