# Supplementary material for: Amino Acid Deficiency Secondary to Continuous Venovenous Hemofiltration in Acute Decompensation of Organic Acidemias: An Anabolic Dead End?
Source: Crit Care Explor. 2026 Aug 3;8(8):e1413. doi: 10.1097/CCE.0000000000001413 (PMC13433117; doi:10.1097/CCE.0000000000001413)
Supplement: Supplementary file 1 [file cc9-8-e1413-s001.pdf]

Amino Acid Deficiency Secondary to Continuous Veno-Venous Hemofiltration in Acute Decompensation of Organic Acidemias: An Anabolic Dead End?

Supplemental digital content

Chloé Grosyeux[1]; Noémie Cammiciotto[2]; Elise Jeannesson[2,3]; Eva Feigerlova[2]; Valentine Villada[1]; Camille Wicker[4]; Michael Levy[5]; Manuel Schiff[6,7]; Apolline Imbard[6,7]; David Coelho[2]; François Feillet[2]; Arnaud Wiedemann[2,9,10]

1. Pediatric Nephrology Department, University Hospital of Nancy, F-54000, Nancy, France.
2. Reference Center of Inborn Errors of Metabolism (ORPHA67872), University Regional Hospital Center. of Nancy, INSERM UMRS 1256 NGERE – Nutrition, Genetics, and Environmental Risk Exposure, Nancy F-54000, France.
3. Department of Molecular Medicine, Division of Biochemistry, University Hospital of Nancy, Nancy, France.
4. Pediatric Inherited Metabolic Diseases department, University Hospital of Strasbourg - Filière G2M -, 1 Avenue Molière, 67200, Strasbourg, France.
5. Pediatric Intensive Care Unit, University Hospital of Strasbourg, 1 Avenue Molière, 67200, Strasbourg, France.
6. Reference center for Inborn Error of Metabolism, Hôpital Necker-Enfants Malades, APHP and Université Paris Cité, filière G2m, MetabERN, Paris, France.
7. Paris-Saclay University, CEA, Département Médicaments et Technologies pour La Santé (DMTS), Gif-sur-Yvette, France.
8. Inserm UMRS_1163, Institut Imagine, Paris, France.
9. Pediatric Intensive Care Unit, University Regional Hospital Center of Nancy, Nancy F-54000, France.
10. Research Center, Sainte-Justine Hospital, University of Montreal, Montreal, QC, Canada.

Table S1. Characteristics of each individual patient

	Gene	Variant 1	Variant 2	Age (days)	Satus	~~IMC~~ BMI (Z-score)	H / W (Z-score)	Carglumic acid	Ammonia		Lactatemia		pH		Protidemia	
									Before	*After*	*Before*	*After*	*Before*	*After*	*Before*	*After*
#1	*PCCB*	c.1283C>T	c.1535G>A	2160	F	-0.7	1.0 / 2.7	Yes	146	38	4.3	3.7	6.988	7.412	42	35
#2	*MMUT*	c.1207C>T	c.1207C>T	3	I	-3.9	-1.4 / 0.6	No	470	94	1.5	6.5	7.254	7.23	30	32
#3	*MMUT*	c.1207C>T	c.1207C>T	480	F	-0.8	-2.0 / -3.2	Yes	86	17	5.9	8.6	7.189	7.346	38	47
#4	*MMUT*	**c.949delA**	c.91C>T	4	I	-1.6	0.1 / 1.1	No	543	48	1.5	7.2	7.267	7.239	45	40
#8	*MMAB*	c.556C>T	c.556C>T	21	I	-3.1	-1.7 / -1.7	No	505	77	0.9	1.9	7.11	7.08	56	50
#9	*MMUT*	c.1843C>A	c.1843C>A	3	I	-2.1	0.1 / -1.4	No	812	696	1	3.5	7.084	7.33	51	40
#5	*PCCB*	c.1283C>T	c.1535G>A	660	F	-4.0	-3.6 / -2.1	Yes	81	10	3.9	5.2	7.205	7.354	36	N/D
#6	*PCCB*	c.1283C>T	c.1535G>A	570	F	-4.0	-3.1 / -1.2	Yes	175	49	1.7	7	6.888	7.379	71	46
#7	*PCCA*	c.1595del	**c.1495del**	3	I	-0.3	0.3 / 0.1	No	1457	82	1.4	2.3	7.254	7.398	54	46

Note. BMI: Body Mass Index; H: height; W: weight; I: initial decompensation; F: followed patient; Before/After: before and after CRRT: continuous renal replacement therapy; in bold: variant not described in the literature

Figure S1. Time-course data for AA of each individual patient.

Note. xLLN: times lower limit of normal

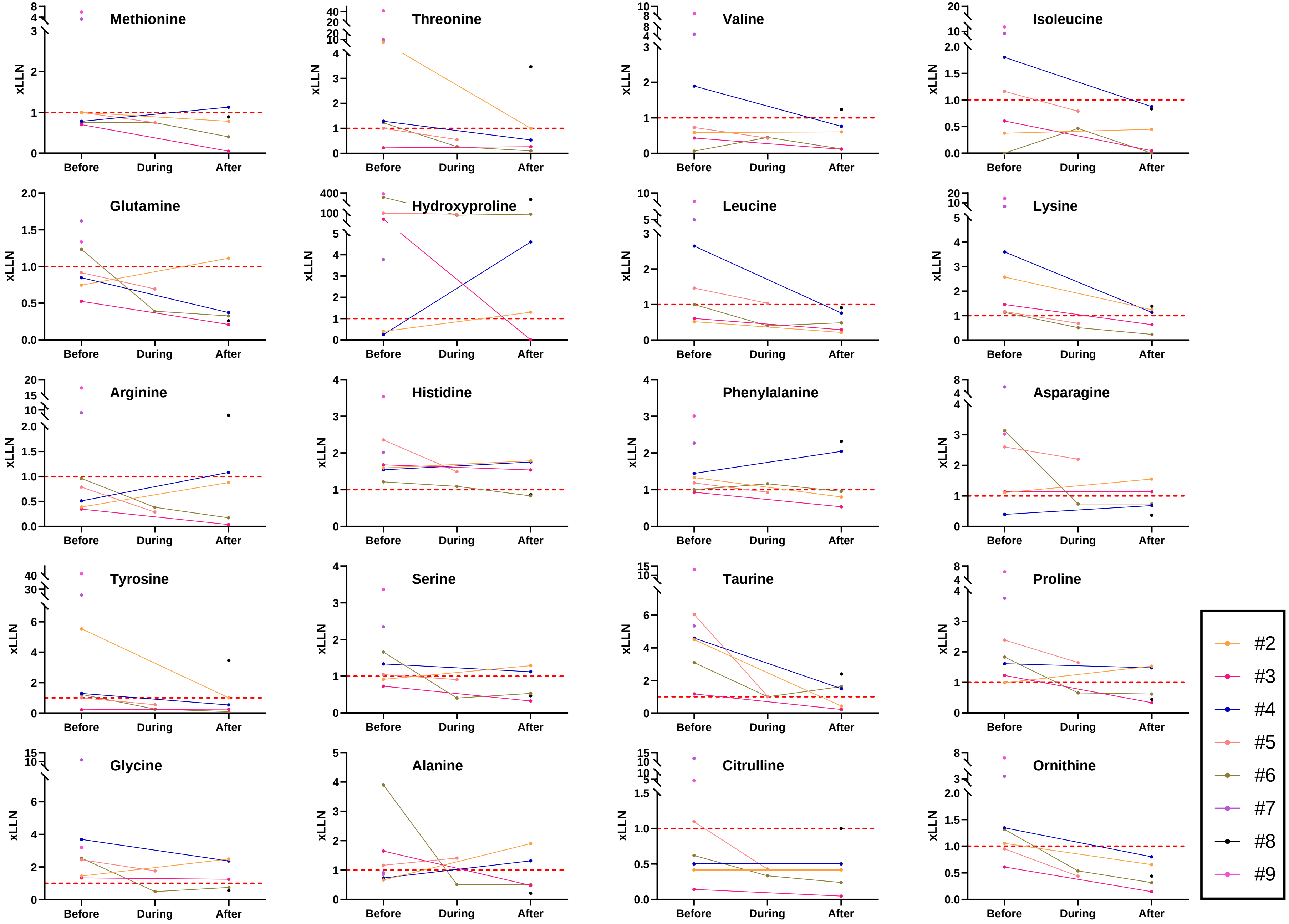
Methionine
Threonine
Valine
Isoleucine
Glutamine
Hydroxyproline
Leucine
Lysine
Arginine
Histidine
Phenylalanine
Asparagine
Tyrosine
Serine
Taurine
Proline
Glycine
Alanine
Citrulline
Ornithine
xLLN
Before
During
After
#2
#3
#4
#5
#6
#7
#8
#9
